# Distributed Detour Routing Scheme for Link Failure with Minimized Overhead in LEO Satellite Networks

**DOI:** 10.3390/s23239590

**Published:** 2023-12-03

**Authors:** Jeongju Im, Jiseung Youn, Soohyeong Kim, Joohan Park, Sejong Lee, Yongseok Kwon, Sunghyun Cho

**Affiliations:** 1 Major in Bio-Artificial Intelligence, Department of Applied Artificial Intelligence, Hanyang University, Ansan 15588, Republic of Korea; dlawjdwn12@hanyang.ac.kr; 2 Major in Bio-Artificial Intelligence, Department of Computer Science and Engineering, Hanyang University, Ansan 15588, Republic of Korea; yjs1104@hanyang.ac.kr (J.Y.); dreammusic23@hanyang.ac.kr (S.K.); kingsaejong@hanyang.ac.kr (S.L.); totoey200@hanyang.ac.kr (Y.K.); 3 Department of Computer Science and Engineering, Hanyang University, Ansan 15588, Republic of Korea; 1994pjh@hanyang.ac.kr

**Keywords:** LEO satellite, distributed routing, flooding, communication overhead, link failure

## Abstract

The mobility of low Earth orbit (LEO) satellites causes the LEO satellite network to experience topology changes. Topology change includes periodic topology change that occurs naturally and unpredictable topology change that occurs due to instability of the inter-satellite link between satellites. Periodic and unpredictable topology change causes frequent topology change, requiring massive communications throughout the network due to frequent route convergence. LEO satellites have limited onboard power because they operate on batteries. The waste of limited satellite onboard resources shortens the lifespan of the satellite, and achieving stable end-to-end transmission is challenging for the network. In this regard, minimizing communication overhead is a fundamental consideration when designing a routing scheme. In this paper, we propose a distributed detour routing scheme with minimal communication overhead. This routing scheme consists of a rapid detour, selective flooding, and link recovery procedures. When a link failure occurs in the network, a rapid detour can detect link failure using only a precalculated routing table. Subsequently, selective flooding searches for the optimal detour point within the minimum hop region and flood to detour point. After link recovery, a procedure is defined to traverse the pre-detour path and switch it back to the original path. The simulation results show that the proposed routing scheme achieves a reduction of communication overhead by 97.6% compared with the *n*-hop flooding approach.

## 1. Introduction

The low Earth orbit (LEO) satellite network can provide global communication services across the Earth through multi-hop routing using inter-satellite links (ISLs). In rural, mountainous, and marine regions where maintenance of the terrestrial network infrastructure is difficult, the LEO satellite network can provide communication services with global coverage through ISL without ground gateway relays. For example, Iridium is a network consisting of 66 LEO satellites, providing a data service with global coverage [1]. SpaceX has recently announced a plan to deploy 12,000 LEO satellites over the next 20 years for global coverage [2]. Therefore, the LEO satellite network can provide global coverage with relatively low latency and is an important part of the next-generation network in providing seamless global coverage [3].

A routing algorithm that considers the characteristics of LEO satellites is necessary to provide global communication services in the LEO satellite network. LEO satellites have mobility, but the ground station is fixed on the ground. The location of the ground station can be a factor in determining the routing path [4]. Data transmission in inter-satellite communication requires pointing, acquisition, and tracking (PAT) processes to establish ISL connections [5]. The ISL connectivity is unstable during the PAT process because of satellite mobility and varying orbital characteristics [6]. Given the instability of ISL, in the absence of a connected link, the satellite may assume a link already exists. This may lead to frequent differences between the actual and the routing topologies. Continuous data transmission over a nonexistent link can cause packet drop and communication interruptions. Frequent topological differences may eventually collapse network reliability. Therefore, topological differences should be considered in designing routing algorithms for LEO satellite networks.

To address the topology differences in LEO satellites, link-state routing has been proposed [7]. In link-state routing, satellites periodically share link-state information with adjacent satellites and flood any changes in the link state throughout the network. Flooding is a method of broadcasting this information to the network when a change occurs in the network topology. Link-state routing allows real-time monitoring of the link state, enabling the immediate handling of topology differences. However, the instability of ISLs intensifies topological differences, leading to frequent flooding, which imposes massive communication overhead on all the ISLs within the network. In particular, LEO satellites have extremely limited processing power and storage because of space and technological constraints [8]. Minimizing the communication overhead of the exchange link state is crucial for the onboard constraints of LEO satellites.

To cope with the communication overhead from routing topology maintenance, a hop-by-hop routing scheme was proposed [9]. In this scheme, satellites calculate the routing table independently and only use the next hop for forwarding. The movement of LEO satellites is predictable, as they move at a constant speed along a predefined orbit. This allows determining the next hop without the need for sharing the link state because the position of the satellite can be determined at any given moment. In a given topology, the optimal next hop can be selected without exchanging information to reach the destination. For example, as the inter-ISL length decreases at high latitudes, the inter-ISL should be selected in the routing path and the intra-ISL is used for transmission at high latitudes because intra-ISL always has the same length, thereby obtaining the shortest inter-ISL in the entire routing path. A hop-by-hop routing scheme is performed without link-state exchange using only the geographic information of the LEO satellite through the LEO constellation information. Therefore, the communication overhead is reduced because there is no need for link-state exchange for route convergence.

The instability of the ISL can easily disrupt the regularity and predictability of the LEO constellation when a satellite or link fails [10]. This is because an unconnected ISL can cause the actual LEO constellation to differ from the predicted LEO constellation. The hop-by-hop routing scheme relies only on the geographical information of the LEO constellation. Because the hop-by-hop routing scheme calculates the shortest routing path within a given topology, it cannot detect link failures caused by ISL instability. A hop-by-hop routing scheme requires a method for sharing link-failure information to resolve unpredictable topological differences. Conventional flooding methods result in high communication overhead and require a more efficient information-sharing approach. Flooding broadcasts the link state throughout the entire network, similar to the *n*-hop flooding approach presented in [11]; *n*-hop flooding reduces the communication overhead by simply limiting the flooding range.

Flooding-based link information exchange incurs unnecessary and redundant link failure information. Link failure information propagated to an unspecified number of nodes within the network generates communication overhead. Wasting a limited onboard processing power of LEO satellites can reduce the resources needed for satellite operation and disrupt reliable end-to-end transmission. The routing path has to be created according to the changing topology while minimizing the link information messages exchanged due to frequent topology changes. Considering the limited processing power of satellites, a routing technique that minimizes link information messages for creating a routing path is needed.

In this paper, we propose a distributed detour routing scheme to minimize overhead. When a link failure occurs in LEO satellite networks, the link failure information should be shared with as many nodes as possible, consuming the least amount of link resources. The proposed scheme finds the best detour point with minimal communication overhead by combining a rapid detour with selective flooding. A preliminary detour path is created using rapid detour packets transmitted through a failed link without any communication overhead. Communication overhead is minimized by selectively flooding the starting point of the generated preliminary detour path to the best detour point within the minimum hop region (MHR) of the packet. The path flooded to the best detour point is combined with the detour path created by the rapid detour to create a pre-detour path. Link-state packets are also exchanged for exclusive link failures to detect link recovery and effectively switch to the original path. This combined distributed detour routing scheme can achieve low delay and packet drop rates with minimal communication overhead.

The main contributions of this study are as follows:

- Rapid detour routing is proposed for a packet encountering a link failure in LEO satellite networks. Rapid detour routing instantaneously detours the failed link and maintains the shortest path without the need for additional path calculation or link-state exchange when a link failure is encountered.
- A selective flooding algorithm is proposed to minimize overhead. The selective flooding utilizes MHR to identify the best detour points without additional hops.
- A link recovery procedure is defined to switch the detour path to the original path. By creating a detour path utilizing rapid detour routing and selective flooding, we can switch back to the original path and minimize end-to-end delay.

The remainder of this paper is organized as follows. In Section 2, related studies are introduced. Section 3 presents the system model of the proposed scheme. In Section 4, the distributed detour routing and recovery schemes are presented. In Section 5, the simulation results are presented. Finally, conclusions are drawn in Section 6.

## 2. Related Works

Related studies are presented in this section on routing algorithms for LEO satellite networks. These algorithms find routing paths in a distributed manner, reducing communication overhead that occurs during the topology update process that changes due to unexpected link failure. We sequentially introduce studies on finding the optimal detour route without updating the routing topology.

Distributed routing configures the topology without global link-state information. Each satellite has an independent routing table. Information exchange between satellites to construct the topology is reduced. A datagram routing algorithm (DRA) was proposed in [12]. In DRA, each satellite independently selected the next hop, with the destination determined based on the logical addresses of the satellites. In [13], the authors proposed the semi-distributed routing algorithm (SDRA). SDRA calculates up to the next hop of the next hop and records this information in the packet header. In [14], the authors proposed local-assisted on-demand routing (LAOR), which is commonly used in mobile ad-hoc networks. LAOR defines the minimum hop region (MHR) from the source to the destination of the satellite. LAOR only finds the routing path when transmission requests it. In [15], the authors proposed a disruption-resistant on-demand routing protocol (DODR) for local repair. DODR improved with the ad-hoc on-demand distance vector routing algorithm. DODR allows routes that change according to frequent topology changes to be detoured through local repair. In [7], the authors proposed on-demand dynamic routing. Regular topological changes are managed through static routing by employing memory-efficient static routing, enabling each satellite to update its routing table without information exchange independently. In the sudden topological changes due to link instability, only the changed link-state information is propagated through the network using on-demand dynamic routing. The distributed routing algorithm does not involve additional information exchange with neighboring satellites, making it ineffective for addressing unexpected link failures. However, sharing information about faulty links for topology reconfiguration can lead to substantial communication overhead.

Studies have been presented to reduce the number of messages shared on networks. In [16], the authors proposed a topology pruning algorithm to reduce overhead during flooding. The orbits of each satellite in the LEO network topology are connected inter-plane. This is a technique for pruning the topology by selecting the shortest inter-plane ISL connecting orbits and connecting each orbit to one inter-plane. In [17], the authors proposed an algorithm that minimized the use of inter-plane links to the destination. To select a routing path, the optimal satellite is chosen that can move into the orbit of the next satellite within the network topology. In [18], the authors proposed a minimum-hop binary tree pruning traversal-based earliest arrival (MHBTEA) routing algorithm. MHBTEA generates the routing path as a binary tree and searches for new paths by pruning the tree according to topology changes. Studies are presented to minimize the number of shared link status messages by clustering satellites. In [19], the authors proposed area-based satellite routing. The network topology is divided into inter- and intra-areas. Each area independently manages its routing table in a distributed manner, and changes in links occurring within an intra-area are propagated only within the same area. In [20], the authors proposed a satellite clustering algorithm. Utilizing game theory, an optimization problem has been formulated to address cluster reliability and management overhead. A hierarchical network architecture has also been proposed, wherein topology changes are managed by other satellites, taking into account the onboard constraints of LEO satellites. In [21], a low Earth orbit–medium Earth orbit–geostationary equatorial orbit (LEO-MEO-GEO), layer structure was produced to reduce the communication costs from LEO routing by dividing into MEO and GEO layers. In [22], the MEO layer collects the link state and calculates the routing table for the LEO layer. This reduces the burden on LEO satellite computing resources for maintaining routing tables. In [23], to increase network reliability, a failure recovery mechanism was applied to the failure cases of the LEO and MEO layers. In the hierarchical architecture, LEO/MEO layers cooperate with each other.

Even a single link change causes all routing tables in the entire network to be updated, wasting the limited power of the satellite. Therefore, studies are conducted to deliver link failure information only to restricted areas. In [24], the authors proposed a fault-tolerant adaptive routing based on a minimal-connected-component model. A faulty and useless node is pre-detoured by fault region boundary diffusion. In [9], the author proposed a survivable routing algorithm. In this algorithm, a vector-based next-hop selection mechanism is used for the routing path calculation. It assigns scores based on whether each path is the shortest or a detour, prioritizing the path with the highest score for selection. When a link failure is detected on a high-priority path, the algorithm switches to the path with the next-highest priority. In [11], the authors proposed a disruption-tolerant routing algorithm. In contrast to traditional flooding methods, this algorithm employs a restricted flooding area with a predefined hop. Nodes that receive flooding are sent in a direction different from the shortest path to detour link failure.

The previous routing algorithms cannot be directly implemented in LEO satellite networks. Distributed routing can reduce communication overhead by maintaining the routing table independently; however, it is vulnerable to link failures caused by topological differences. With the massive number of satellites in LEO constellations, clustering and topology pruning may incur substantial communication overhead from frequent information exchange in the presence of topological differences and link failures. Hierarchical structures can share the burden of LEO satellites on other layers but cannot address communication delays between layers. Fault link information shared only to restricted areas is using the flooding method. Flooding can be used to generate an optimal detour path instead of generating massive communication overhead throughout the network. Distributed routing using the *n*-hop flooding method incurs a relatively small communication overhead but cannot create the shortest detour path. Considering the onboard power limitation of LEO satellites and detour path delay, we propose a distributed detour routing scheme to minimize the communication overhead and delay of the detour path.

## 3. System Model

In this section, we describe the system model considered in this study and present the conventional LEO satellite constellation and ISL interface. In the LEO constellation, MHR is defined to find the shortest path. The hop-by-hop routing scheme determines the shortest path with the lowest communication overhead. When a packet encounters a link failure, the flooding method is utilized to share the link-state information. Thus, to minimize overhead during flooding, we define the communication overhead required for flooding.

### 3.1. LEO Satellite Constellation

In the LEO constellation system, we focus on a conventional polar orbit with an inclination of approximately $90^{\circ }$. An LEO satellite network is defined as a graph G=(V,E), where *V* is the set of satellites and *E* is the set of ISLs. A typical N×M constellation comprises *N* orbits and each orbit contains *M* satellites. The orbital planes are separated from each other by the same angular distance of 2π/(2×N). The satellites in the orbital plane are separated from each other by the same angular distance of 2π/M. Considering zero eccentricity for each orbit results in the satellites revolving in circular orbits. Following other studies, we considered the usual type of ISLs: (i) each satellite has two intra-plane ISLs (links with adjacent satellites in the same orbit plane) and (ii) two inter-plane ISLs (links with satellites in the adjacent orbit).

**Figure 1:**
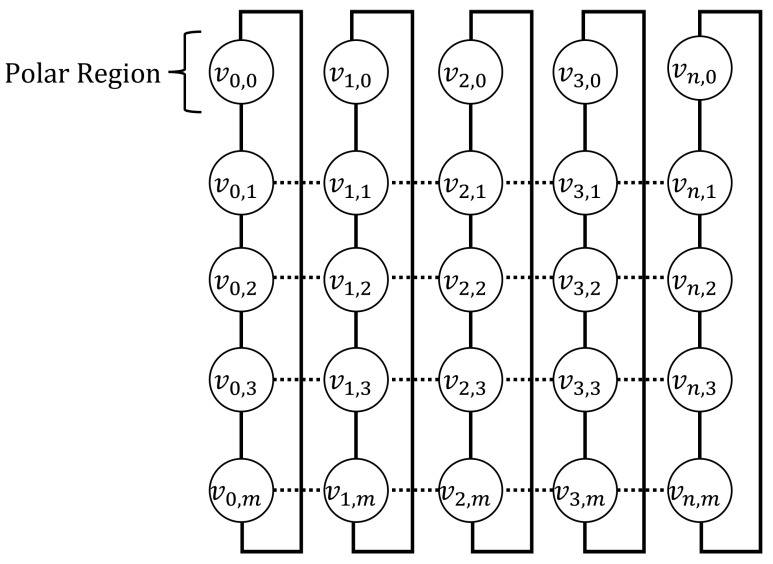
LEO satellite polar constellation.

The topology of the LEO satellite constellation is shown in Figure 1. The dashed line represents the intra-plane ISL, and the dotted line in Figure 1 represents the inter-plane ISL. The location of each satellite can be represented by $\left(v_{n,m}\right)$; *n* is the plane index and *m* is the satellite index, where n∈{0,1,⋯,N} and m∈{0,1,⋯,M}. Each satellite has four directional ISLs, denoted by $E=\{e_{n,m}^{\mathrm{dir}}|\underset{\forall n,m}{\mathrm{dir}}\in \left\{\mathrm{up},\mathrm{left},\mathrm{down},\mathrm{right}\right\}\}$. LEO satellites in the polar region cannot establish an inter-plane ISL with adjacent satellites. The polar constellation has a seam because of the opposing relative velocities between the satellites. Thus, cross-seam ISL connections are unavailable. Therefore, satellites within the polar region have two ISLs and satellites near the seam have three ISLs.

### 3.2. Minimum Hop Region (MHR)

**Figure 2:**
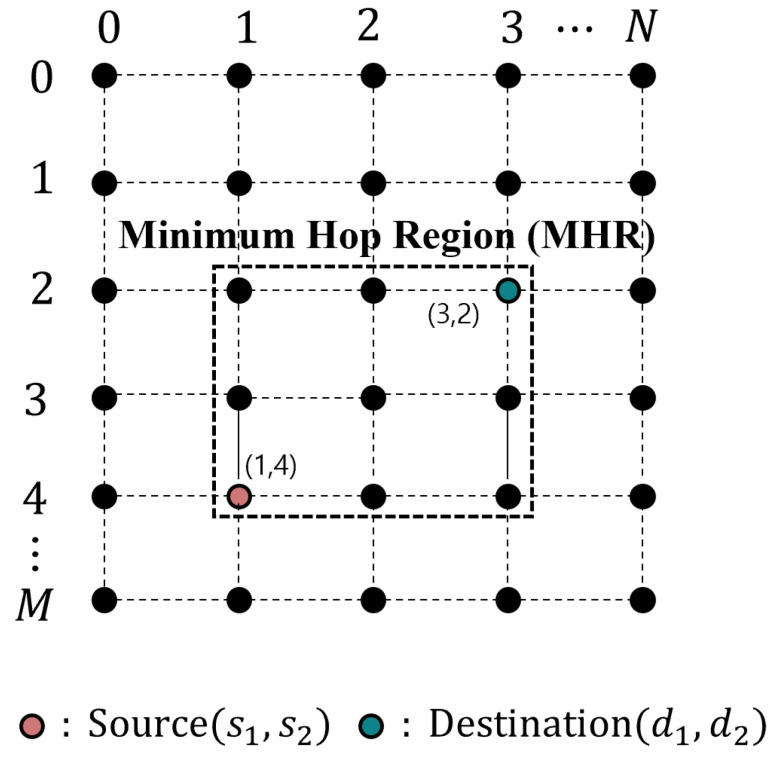
Minimum hop region in LEO satellite network.

The concept of MHR was originally proposed in [14] as a region defining a set of paths with minimal hops between the source and destination to restrict request messages. Given the network topology G=(V,E), MHR is defined by the generated sub-graph $G^{\prime }=\left(W,E^{\prime }\right)$. Let us assume that the source satellite ID is $v_{s_{1},s_{2}}$ and the virtual coordinates are $\left(s_{1},s_{2}\right)$. The destination satellite ID is $v_{d_{1},d_{2}}$ and the virtual coordinates are $\left(d_{1},d_{2}\right)$. In other words, the minimum hop region for packet *k* is $MHR^{k}=\left\{\left(v_{s_{1},s_{2}},v_{d_{1},d_{2}}\right)|\left(v_{s_{1},s_{2}},v_{d_{1},d_{2}}\right)\in W,v_{s_{1},d_{1}}\in M_{x},v_{s_{2},d_{2}}\in M_{y}\right\}$, where $M_{x}$ and $M_{y}$ denoted the MHR edge. The *x*-axis of the MHR edge is $M_{x}$ = $\left[x_{\min},x_{\max}\right]$ with $x_{\min}=$ min$\left\{s_{1},d_{1}\right\}$ and $x_{\max}=$ max$\left\{s_{1},d_{1}\right\}$. The *y*-axis of the MHR edge is the same as that of the *x*-axis. Subsequently, MHR is defined as shown in Figure 2.

MHR always does not always have a rectangular shape. If the coordinates of either the *x*-axis or *y*-axis of the source $v_{s_{1},s_{2}}$ or destination $v_{d_{1},d_{2}}$ in the graph are the same, then the shape of MHR is a line. If $s_{1}$ and $d_{1}$ have the same coordinates, then MHR is along a vertical line in the same orbit. Conversely, if $s_{2}$ and $d_{2}$ have the same coordinates, then the MHR is a horizontal line. For the routing path to have the minimum number of hops, a packet must be transmitted within the MHR. But, if MHR has a line shape, no detour paths exist within MHR. In the proposed scheme, to determine the optimal detour point in case of a link failure, the MHR must be of a rectangular shape.

Therefore, we extend the linear MHR to form a rectangular shape. When the MHR has a vertical line shape in the same orbit, it is extended in the seam-away direction. When the MHR has a horizontal line shape, it is extended toward the pole. Inter-ISL is shorter at high latitudes and the MHR is extended toward the poles. However, if the extended area crosses the polar boundary, the MHR is extended in the opposite direction to maintain the polar boundary.

### 3.3. Hop-by-Hop Routing Scheme

Routing is conducted within the defined MHR between the established source and destination. In the grid-mesh topology, the minimum-hop path between the source and destination satellites is formed along the edge of the defined MHR. The shortest path follows this edge. In this case, the criterion for selecting the optimal direction is the length of the ISL between the two satellites. The length of the intra-plane ISL $L_{v}$ is fixed and calculated as follows [12]:

(1) $$L_{intra}=\sqrt{2}R\sqrt{1-\cos\left(\frac{2\pi }{M}\right)},$$

where *R* is the orbital radius. The length of the inter-plane ISL, denoted as $L_{inter}$, varies according to the latitudes of the two neighboring satellites, calculated by

(2) $$L_{inter}=\alpha \times \cos\left(\mathrm{lat}\right),$$

where lat is the satellite latitude the inter-plane ISL is connected to (the constellation phase factor is zero). α is calculated as follows:

(3) $$\alpha =\sqrt{2}R\sqrt{1-\cos\left(\frac{2\pi }{2\times N}\right)}.$$

Considering the geographical characteristics of the Earth, the lengths of the inter-ISLs connected at higher latitudes are shorter than those near the equator. However, the intra-ISLs connecting satellites in the same orbit have the same length for all satellites. Therefore, if the latitude of the current satellite is higher than that of the destination satellite, the shorter-length inter-ISL is selected as the primary direction. However, if the latitude of the current satellite is lower than that of the destination satellite, the intra-ISLs are selected for the primary direction. This process continues until the satellite reaches the same latitude as that of the destination. Thus, the next hop is selected to minimize the total length of all the ISLs constituting the path without exchanging information between the satellites.

When the routing path is through the polar region, inter-ISL connections are not possible in polar regions. To utilize a shorter inter-ISL, the intra-ISL is primarily used up to just before the polar boundary to transmit up to the same plane as that of the destination. Once the satellite reaches the polar boundary, the shortest available inter-ISL is selected as the primary direction. Therefore, the hop-by-hop routing scheme utilizes only the geographic location information of the two satellites. The path with the shortest ISL is chosen as the next hop. The pair consisting of the destination and direction of the next hop is matched and stored in the routing table for further use. In the case of link failure, a strategy is required needed to reduce the number of dropped packets. To handle potential link failures, both primary and secondary next-hop directions are precalculated. If the primary direction is unavailable, the forwarding direction is determined using a precalculated secondary direction. This provides a backup plan for routing against unexpected network problems.

### 3.4. Communication Overhead for Flooding

In the next step, link failure information must be communicated to other nodes in the network to avoid passing through a failed link. Both the link-state and hop-by-hop routing schemes utilize the flooding technique to broadcast link-state information. The flooding method broadcasts the link-state data to every node in the network. Thus, all available links are utilized to transmit the link-state information, which requires the recalculation of the routing table. In defining the communication overhead, the links used to broadcast link-state information are considered. Subsequently, to minimize the communication overhead during flooding, the solution is obtained by minimizing the objective function, as follows:

(4) $$Minimize:\sum_{v\in V}f\left(v_{\left(n,m\right)},v_{\left(n^{\prime },m^{\prime }\right)}\right)\times e_{\left(n,m\right),\left(n^{\prime },m^{\prime }\right)},$$

(5) $$s.t.\;f\left(v_{\left(n,m\right)},v_{\left(n^{\prime },m^{\prime }\right)}\right)\in \left\{0,1\right\},$$

(6) $$e_{\left(n,m\right),\left(n^{\prime },m^{\prime }\right)}\in \left\{0,1\right\},$$

where $f(v_{\left(n,m\right)},v_{\left(n^{\prime },m^{\prime }\right)})$ denotes flooding from node $v_{\left(n,m\right)}$ to $v_{\left(n^{\prime },m^{\prime }\right)}$. If node $v_{\left(n,m\right)}$ floods the link state to node $v_{\left(n^{\prime },m^{\prime }\right)}$, the value is one; otherwise, it is zero; $e_{\left(n,m\right),\left(n^{\prime },m^{\prime }\right)}$ denotes the link between nodes $v_{\left(n,m\right)}$ and $v_{\left(n^{\prime },m^{\prime }\right)}$ in *E*. If $e_{\left(n,m\right),\left(n^{\prime },m^{\prime }\right)}$ is used during flooding, it has a value of one; otherwise, it is zero. According to Equation (4), overhead is minimized when a link failure occurs in LEO satellite networks.

## 4. Proposed Routing Scheme

To reduce the communication overhead when a link failure occurs in an LEO satellite network, we propose a distributed detour routing scheme. Link failures can increase the delay in the routing path and create incomplete paths, potentially leading to higher packet drop rates. Therefore, the proposed scheme introduces algorithms aiming to reduce both the packet drop rate and routing path delay with minimal communication overhead.

### 4.1. Distributed Detour Routing Scheme

**Figure 3:**
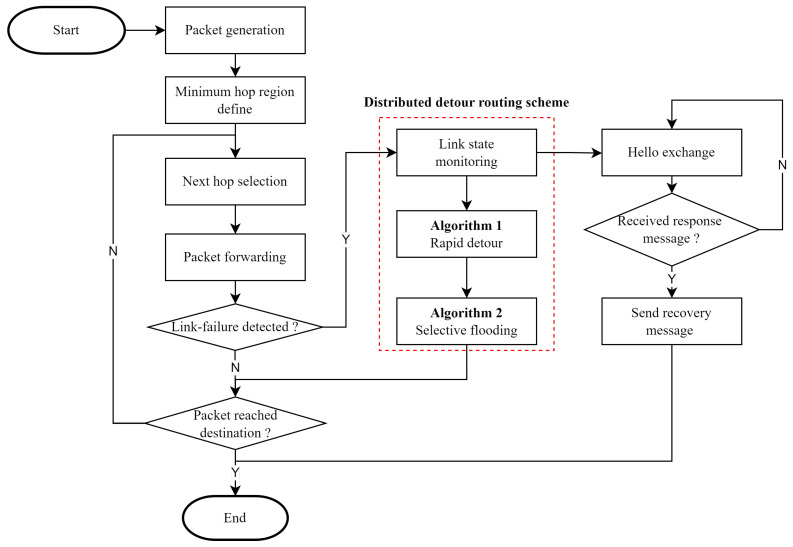
The proposed detour routing scheme.

The distributed detour routing scheme comprises rapid detour routing, selective flooding, and a link recovery process. From a network-wide perspective, the distributed detour routing scheme is executed after a link failure, as illustrated by the red dotted box in Figure 3. In the proposed scheme, we consider only inter-satellite routing.

Upon packet creation, the minimum hop region is automatically established. Subsequently, the next hop is selected to ensure the shortest path, and the packet is transmitted through the ISL accordingly. Packet forwarding is repeated until the packet reaches its destination. The proposed scheme receives a response message from the next hop in each packet-forwarding process. If a response message is not received within a predefined time, then the ISL is considered to have experienced a link failure.

We assumed that link failure occurs due to instability in the PAT process for connecting the ISL. When a link failure is detected as shown in Figure 3, the distributed detour routing scheme is utilized. The first step in the proposed scheme is link-state monitoring, which detects a link recovery through hello exchanges. Link-state monitoring continues until link recovery is detected. In the next step, we form a pre-detour path using both the rapid detour and selective flooding algorithms shown in Figure 3:

- Rapid detour is an algorithm that reroutes the original packet, which failed to transmit to its destination. This detouring is accomplished using only the precalculated routing table and geographical information without incurring any communication overhead to transmit link information. Consequently, Algorithm 1 only utilizes the given prior information to identify a rapid detour direction from the routing table to detour the link failure. A detailed description of Algorithm 1 is discussed in Section 4.2.
- Selective flooding is an algorithm that establishes a pre-detour path within the MHR of the original packet so that the satellite can detour the link failure. Unlike the conventional flooding mechanism, selective flooding has a specified destination. Selective flooding aims to find a detour point that can cover the entire MHR of the original packet with minimal communication overhead. Therefore, in Algorithm 2, determining the destination for selective flooding requires finding the optimal detour point. A detailed description of Algorithm 2 is discussed in Section 4.3.

The pre-detour path created by the distributed detour routing scheme detours link failures without any hop loss, with minimal communication overhead. When link recovery is detected through link-state monitoring as shown in Figure 3, the recovered node sends a recovery message along the pre-detour path to revert the routing path. The link recovery procedure is described in Section 4.4.
**Algorithm 1:** Calculating rapid detour direction
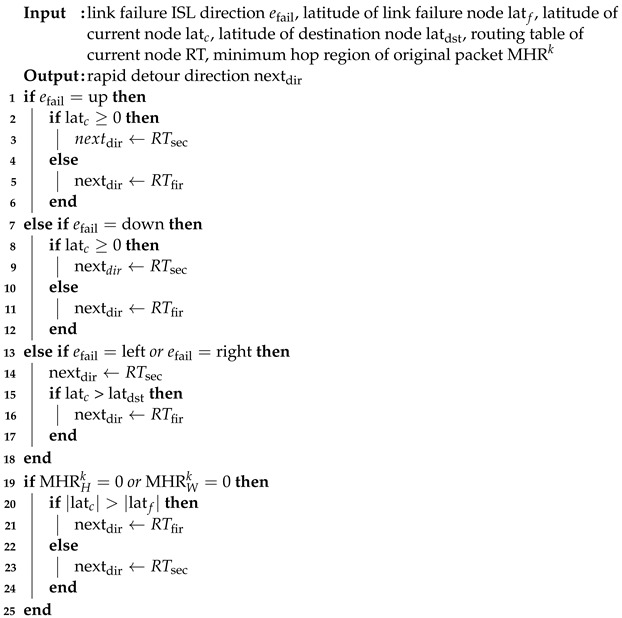

**Algorithm 2:** Calculating selective flooding path
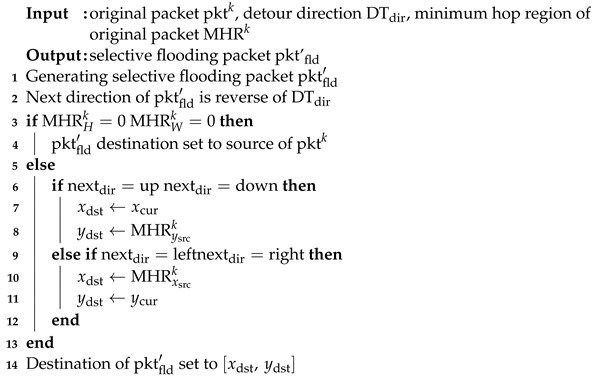


### 4.2. Rapid Detour Routing

Packets that encounter a failed link are rerouted without additional path computation through rapid detour routing. The first step in rapid detour routing is to forward the original packet in the secondary direction from the link failure. During this process, a detour field containing the failed link information is added to the header of the original packet. The detour field indicates that a packet has taken a detour because of encountering a failed link. Therefore, only a 1-bit detour identifier is added to the packet header.

The node receiving the detour packet generates a detour table. The detour table records the destination of the detour packet, the direction of the failed link, and the next direction calculated for the next transmission. The node calculates the detour packet sent in the secondary direction using the rapid detour direction calculation algorithm, as described in Algorithm 1. Lines 1–17, indicate the link failure that has occurred in each direction. In line 19, $MHR_{H}^{k}$ and $MHR_{W}^{k}$ denote the height and width of MHR, respectively. Therefore, if the height or width is zero, the shape of MHR is a line. Algorithm 1 utilizes the latitude of the satellite and the MHR information of the packet for computation without additional information exchange. Since it is calculated from a rapid detour point without an iterative process, Algorithm 1 always has a time complexity of O(1).

The satellite determines the detour direction based on its current latitude and information regarding the link failure node (latitude and failure direction) included in the header of the detour packet. Rapid detour routing detours packets using only the precalculated routing table, without additional computing. Thus, the packet is transmitted by selecting either the primary or secondary direction, and both directions must be within the MHR region.

**Figure 4:**
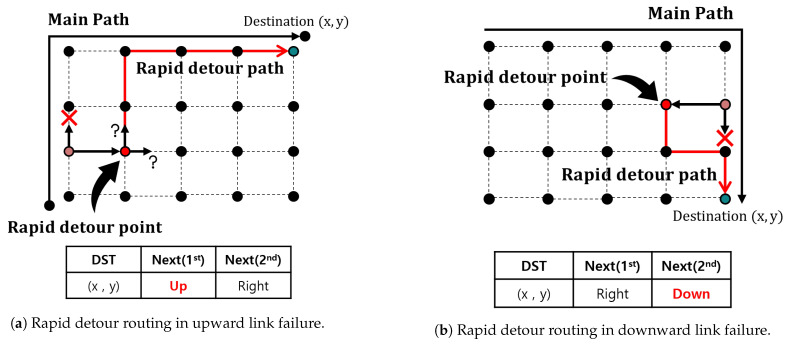
Rapid detour routing in different link failure directions.

Figure 4 shows a rapid detour routing process when link failure occurred in the Northern Hemisphere, and Figure 4a shows a rapid detour process link failed in the upward direction. Packets that encounter link failure are detoured in the secondary direction. The node that receives the detour packet in the secondary direction becomes the rapid detour point, as indicated by the red node in Figure 4a. At the rapid detour point, the next direction is chosen between the primary and secondary directions to determine the detour path. In this case, proceeding in an upward direction results in shorter inter-ISL lengths. Thus, the detour packet is forwarded in the primary direction, which is the upper direction and has a shorter inter-ISL. Figure 4b illustrates a case of rapid detour routing when link failure has occurred in the Northern Hemisphere in the downward direction. At the rapid detour point, if the detour is in the primary direction, the path would once again pass through the link failure. Therefore, from the rapid detour point, the packet is rerouted in the secondary direction to reach its destination. Comparing the two cases shown in Figure 4a,b, the rapid detour point in an upward link failure has two options: the primary or secondary direction. However, in the case of a downward link failure, the secondary direction must be selected to avoid loops.

In the Southern Hemisphere, the routing approach is opposite to that of the Northern Hemisphere, such that the upward direction is the secondary direction and the downward direction is the primary direction. In the Southern Hemisphere, the downward direction of the ISL is toward high latitudes. Therefore, the route is heading in a direction opposite to that of the Northern Hemisphere. If a link failure occurs in the left or right direction, the next hop decision is made within the MHR. To determine which direction to use, the rapid detour algorithm considers the latitude of the current node and the location of the failed link. If the latitude of the current node is higher than that of the failed link, the primary direction is selected. Conversely, if the latitude of the current node is lower than that of the failed link, the secondary direction is chosen.

The rapid detour process is repeated until the original packet reaches its destination. After transmission ends, the detour table compares the destination of other packets with the destination in the detour table. If the destination in the detour table matches the destination of the packet, the packet is forwarded to the next hop specified in the detour table. Rapid detour routing detours failed links instead of using the original routing table. To effectively minimize packet drop within a network, the instant detouring of packets from any failed link is required. By implementing rapid detour routing, networks can decrease the overall packet drop rate, thereby ensuring reliable data transmission.

### 4.3. Selective Flooding within Minimum Hop Region

As the rapid detour path results in more hops compared to the shortest path within the MHR, establishing a pre-detour path is necessary. Instead of flooding all the nodes within the MHR, selective flooding discovers an optimal detour point to directly send the link failure information. Nodes between the rapid detour point and the detour point receive the link failure information and generate a detour table. This detour table works similarly to rapid detour routing in redirecting the pre-detour packets that can pass through the failed link.

The proposed selective flooding algorithm selectively propagates link failure information to the optimal detour point. Unlike traditional flooding methods, link information is not transmitted to all nodes within reach but is only sent along the specific detour point within the MHR. Consequently, the key objective of the selective flooding algorithm is to find a detour point. To determine the detour point for selective flooding, the packet uses its MHR region. In this study, the shortest path used in the distributed routing is identical to the MHR edge of the packet. The optimal routing path is the same as the edge of the MHR of the packet. Therefore, packets with the same MHR have to pass through the MHR edge. Finally, the detour point becomes the MHR edge of the rapid detour routing packet.

**Figure 5:**
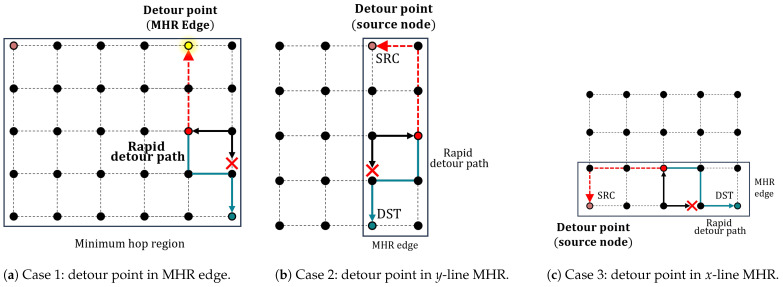
Finding detour points in different MHR shapes.

The MHR edges $M_{x}$ and $M_{y}$ correspond to the x,y coordinates of the source and destination, respectively, and the link failure information is sent to the detour point. The messages that are selectively flooded to the detour point instruct each node to create a detour table. As shown in Figure 5a, the red dotted line is a selective flooding path from the rapid detour point (red dotted node). The rapid detour sets the next-hop field in the detour table using the calculated result for the next hop. However, the detour table created by selective flooding sets the previous node as the next hop. This means that the path of the selective flooding transmitted up to the detour point achieves the same effect as the pre-detour path. When packets pass through the link failure node in the MHR region, they must take the pre-detour path to reach the destination using the detour table. When packets follow their original path, the destination is compared with that in the detour table. If the destinations match, the packets are detoured using the detour table.

When the MHR shape is not a square, its edge and the original path of the packet do not intersect. Therefore, the optimal detour point cannot be determined. In this scenario, the source node of the original packet is used as the destination for selective flooding, as shown in Figure 5a,b. The selective flooding path calculation is outlined in Algorithm 2. The calculation for the selective flooding path requires only the packet information with MHR. Without the iterative operations, the time complexity of Algorithm 2 is O(n), increasing in accordance with the number of hops through which the selective flooding is transmitted. Depending on the location of the link failure, optimal detour paths can be created without selective flooding. When a link failure occurs at the MHR edge where the source node is located (i.e., the source edge), an optimal detour path can be generated without selective flooding.

For example, let us assume that the source node is located at a lower latitude and the destination node is located at a higher latitude. In this case, the source node is forwarded through the intra-ISL for the shortest path to utilize the higher-latitude inter-ISL. To find the shortest pre-detour path, the detour should occur from the point with the shortest inter-ISL and not from the source. Therefore, selective flooding is not required if the link failure is located at the source edge and is upward in the Northern Hemisphere or downward in the Southern Hemisphere.

Similarly, let us assume that the source node is located at a higher latitude than the destination node and that a link failure has occurred at the source edge. In this case, if selective flooding is used for the pre-detour, the packet is forwarded through an inter-ISL at a lower latitude than that of the source node. If the source node is located at a higher latitude than the destination node and link failure occurs at the source edge, selective flooding is not performed. In both scenarios, the optimal detour path is obtained using the path determined through rapid detour routing. This represents the detour path established only by the rapid detour, without incurring any communication overhead from flooding.

When a flooded packet encounters a link failure during selective flooding, the flooding packet detours using the same method as the rapid detour. The selective flooding packet also contains information on the source and destination, such as the data packet. The destination location is calculated using Algorithm 2. In Figure 5a, the red dotted line represents the path of selective flooding. Assuming that the link failure has occurred in the upward direction from the rapid detour point, the packet is rerouted in either the left or right direction to reach the detour point.

By implementing selective flooding and rapid detour routing, we created a pre-detour path for all transmitted packets within the MHR. This allows for detour link failure with minimal communication overhead and without consuming additional computing resources. Selective flooding generates flooding messages by the size of MHR. We assume a length of MHR edge $MHR_{l}$ in which link failure occurs. With the rapid detour, selective flooding only floods a message to the detour point from the rapid detour point. Therefore, the scenario that generates the most flooding messages corresponds to $MHR_{l}-1$. In certain cases, a pre-detour path can be established through rapid detour routing alone. This approach is highly effective in minimizing the communication overhead in the event of link failure. By utilizing rapid detour routing with selective flooding, a pre-detour path can be established with minimal communication overhead, thereby saving satellite onboard resources and power.

### 4.4. Link Recovery Process

An optimal routing path can be determined by continuously monitoring link connectivity. This monitoring includes link failures and recovery. Link-state monitoring is initiated immediately after a link failure. The link recovery process is repeated until the link is restored during the distributed detour routing. Link recovery is identified differently from link failure. Although link failures are detected after sending a packet while awaiting a response message, link recovery is confirmed through periodic hello messages.

The process of confirming link recovery follows the flow of link-state monitoring, as shown in Figure 3. If a response is received from the failed link, a link recovery process is initiated. The link recovery process aims to restore the original path by undoing the pre-detour path caused by link failure. Rapid detour routing and selective flooding generate and update the detour table using detour and flooding packets. Thus, the recovered node sends a link recovery message and deletes the information from all detour tables that were established while detouring the previously failed link. Sharing a link recovery message can result in excessive communication overhead. To mitigate this problem, we defined the link recovery process using a distributed detour routing scheme.

The process of sending a link recovery message is executed using the same procedure as that used for constructing the pre-detour path in the distributed detour routing scheme. The link recovery message includes information about the failed link and the destination information of the original packet. The node compares the destination and failed link information stored in the detour table and deletes this information from the detour table. For rapid detour routing, the link recovery process sends a recovery message in the same direction as that calculated using Algorithm 1. After receiving the recovery message, nodes update their detour tables to revert to their original paths. The pre-detour path is composed of both rapid detour routing and selective flooding paths. The link recovery process is performed in the same manner as in rapid detour routing for selective flooding. The link recovery process involves transmitting messages along a precalculated detour path, thus not requiring additional calculations or iterative operations. Consequently, the time complexity is O(n), determined by the number of hops.

### 4.5. Loop Avoidance

This section presents techniques to avoid routing loops in distributed routing by utilizing rapid detours and selective flooding instead. Lacking a global view of routing topology often leads to routing loops and significant packet losses in distributed routing. Therefore, it is important to implement a loop avoidance mechanism to circumvent potential loops caused by link failures.

**Figure 6:**
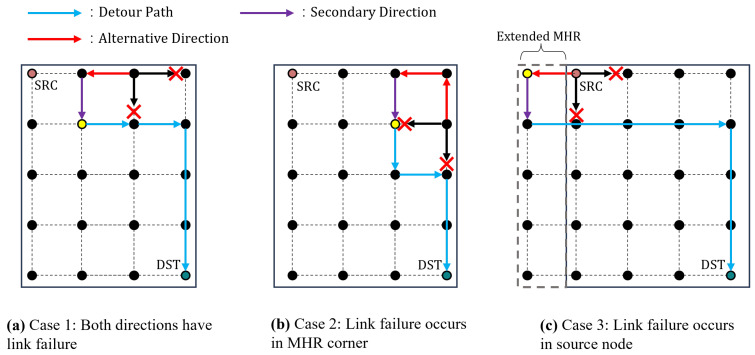
Different loop avoidance strategy by link failure location.

The proposed detour algorithm uses only primary and secondary candidate hops in a distributed hop-by-hop routing scheme. We assumed that packets are forwarded along the MHR edge when both candidate directions are unavailable. In this scenario, an alternative direction is utilized. The alternative direction is opposite to the primary direction, as shown in Figure 6a. Routing looping may occur if the packet returns to its previous transmission path. To prevent looping, packets forwarded in the opposite direction also use a rapid detour to reach their original destination. In Figure 6a, the yellow node represents the rapid detour point. In case of a rapid detour occurring at a node before the detour point, the packet is sent back to the node where the link failure occurred, which can lead to an infinite loop. Therefore, as depicted in Figure 6a, the packet utilizes both alternative and secondary directions (each once for forwarding). The packet then breaks out of the loop and is transmitted to its destination.

As shown in Figure 6b, the packet returns to the corner of MHR. MHR corner ISLs have only primary and secondary directions. If the packet is forwarded outside the MHR of the original packet, additional communication overhead will occur to create a pre-detour path. To minimize communication overhead and escape the loop, a pre-detour path should be established within the MHR region. Unlike the cases in Figure 6a,b, the packet is transmitted twice in the secondary direction. After transmitting through two hops in the secondary direction, the next forwarding direction is determined based on the rapid detour. Figure 6b shows the packet path in the Northern Hemisphere. In the Southern Hemisphere, the path of the packet also forms the same corner shape at high latitudes. Regardless of the hemisphere, the same loop avoidance strategy is applied.

As shown in Figure 6c, the packet returns to a source node. Because all available ISLs in the MHR region are not usable, the MHR is extended to allow detours. In Figure 6c, if a link fails in the right direction, the MHR extends toward the left. Similarly, if a link failure occurs in the upward or downward direction, the MHR is extended in the opposite directions. In the extended MHR, the returned packet undergoes the same procedure as a detour packet. That is, the node that receives the returned packet forwards it through a detour path. Because the source node has already received the link failure information from the returned node, selective flooding is unnecessary.

In case the extended MHR is in the seam or polar region, extending the MHR in the direction opposite to the primary direction can lead to packet drop. If the MHR extends into the seam or polar region, the MHR extends in the direction opposite to the secondary direction of the detour. When the MHR extension reaches the seam, the cross-seam ISL remains unconnected. Extending in the direction opposite to the primary direction may not be feasible. When the direction opposite to the primary cannot be connected, the opposite direction of the secondary direction is selected for the detour. If both the primary and secondary direction extensions are impossible, then all four links are unavailable.

In all three cases, the direction of the returned packet is determined by the rapid detour direction. Additionally, because the returned packet contains information on the failed link, there is no need for additional selective flooding.

## 5. Performance Evaluation

In this section, the performance of the distributed detour routing scheme is presented. The main performance metrics for link failure include end-to-end delay and communication overhead. Furthermore, the extra delay and number of hops for the detoured packet path are investigated using the detour algorithm. Finally, the ISL utilization of the detour path is examined with regard to unstable links.

### 5.1. Simulation Setup

The simulations were conducted on an Iridium-like LEO satellite network to evaluate the performance of the proposed distributed detour routing scheme. The Iridium satellite network comprises 66 satellites with six orbits [1]. That is, each orbit has 11 satellites. Each satellite has four ISLs: two intra-ISLs and two inter-ISLs. The LEO satellites in the polar and seam regions have three ISLs and two ISLs, respectively. The LEO satellite altitude was 780 km and its inclination was $86.4^{\circ }$. Similar to related studies on LEO satellite networks [7], we set the polar boundary of each hemisphere to $70^{\circ }$. We transmitted each packet at a constant rate. We assumed the ISL rate was set to 100 Mbps. This conservative value is achievable with current laser communication terminals [9]. Packets were randomly generated across the entire network topology following a uniform distribution and were transmitted by selecting paths according to hop-by-hop routing. The simulation parameters are listed in Table 1.

In our satellite network, the number of failed ISLs was determined by the link failure rate and was randomly selected from 222 ISLs. Link failure rates follow a uniform distribution in accordance with a uniform traffic distribution. Subsequently, these ISLs were designed to fail at random times within the simulation time. When using a laser module for ISL, the link setup time ranged from a few seconds to tens of seconds [25]. Therefore, link recovery was ensured to occur within a maximum of one minute after the beginning of link failure.

The performance of the proposed distributed detour routing scheme was compared with those of two benchmark schemes. The detailed benchmark schemes are as follows:

- Orbit prediction shortest path first (OPSPF) [7]: In this scheme, the periodic exchange of hello messages was used to detect link failure. After detecting a link failure, similar to the link-state algorithm, the node floods the information to all other nodes in the network for the routing table convergence. When a recovered link is detected, the entire network is flooded with the same process used during link failure to refresh the routing table.
- Disruption tolerant distributed routing (DTDR) [11]: In this scheme, the node that detects a link failure utilizes an *n*-hop flooding strategy, where flooding is restricted to a defined area. Nodes that receive the *n*-hop flooding update their alternative routing tables based on the link failure information. The alternative routing table prioritizes using the secondary direction over the primary direction for paths leading to the destination of the detour link failures. In the simulation, we adopted the three-hop flooding technique proposed in the referenced paper.

### 5.2. Communication Overhead from Flooding Comparison

In this simulation, we compared the communication overhead incurred when sharing link failure information with other nodes following link failure. Specifically, we considered only messages that shared routing information, excluding data transmission. The number of communication overheads is given by Equation (4). In this paper, since the number of flooding messages is equivalent to the communication overhead, the number of flooding messages is calculated as follows:

(7) $$\sum_{i=0}^{N}M_{i},$$

where $M_{i}$ denotes the number of flooding messages generated at each stage, and the range of flooding is *N*. In this experiment, the *n*-hop flooding in DTDR initially propagates three messages, i.e., $M_{0}=3$, and since the flooding range is three, N=3. From the subsequent steps, the flooding starts from the node that received the message in the previous step, i.e., $M_{i}=3\times M_{i-1}$. Conversely, the proposed selective flooding uses a flooding range that extends from the rapid detour point to the detour point in terms of hop count. Since only a single link is used for flooding at each hop, $M_{0}=1$ and $M_{i}=1$ throughout. The transmit power used to send flooding messages consumes onboard power in proportion to the number of messages. The onboard power of each satellite used for flooding is proportional to the number of transmitted messages. In other words, typical flooding consumes the transmit power for three links, excluding the link that received the message.

**Figure 7:**
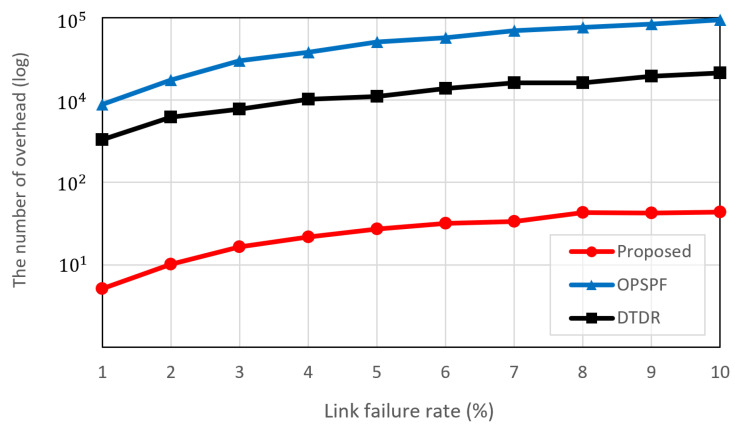
Average communication overhead from flooding according to link failure rate.

Figure 7 shows the communication overhead from flooding based on the link failure rate. OPSPF results in the highest communication overhead. This is because the flooding of the entire network is proportional to the number of ISLs connected to the LEO satellite network. As a result, a single link-state change causes a huge communication overhead. DTDR utilizes the *n*-hop flooding technique, which limits flooding to a restricted area. Since three-hop flooding was used in this experiment, all nodes located within three hops are flooded based on the node that detected link failure. However, it still generates duplicate flooding messages for an unspecified number of nodes. The proposed scheme outperforms both OPSPF and DTDR. This is because the proposed scheme searches for the optimal detour point within a predefined MHR that intersects the paths of the packets. Subsequently, the proposed scheme only communicates link failure information to the nodes at the detour point and along the selective flooding path. This approach eliminates redundant and unnecessary information exchanges, resulting in a significant reduction in communication overhead. The *n*-hop flooding consistently floods link-state information whenever a packet encounters a link failure. The proposed scheme, however, demonstrates a significant reduction in the number of overheads by either not performing selective flooding or by flooding less than the number of routing hops, depending on the location of the link failure. In particular, for the highest failure rate, the proposed scheme produces only 0.48% of the communication overhead compared to OPSPF.

The number of ISLs present in the constellation influences communication overhead. The number of flooding messages in the proposed algorithm is determined by the value of *N* in Equation (7). Additional experiments were conducted to determine the extent of the difference in flooding messages in constellations with more satellites than the Iridium constellation. Therefore, to assess the impact on communication overhead, we varied the number of satellites comprising the constellation. The number of satellites chosen to form the constellation was based on existing commercial satellites, specifically those with a polar constellation configuration similar to that of the simulation environment. Each constellation consisted of 8×16, 6×58, and 12×49 satellites for Telesat, SpaceX, and OneWeb, respectively [26]. Table 2 shows the amount of overhead for different LEO constellations. In each constellation, the link failure rate was based on the median value of 5% used in the previous experiments.

Both OPSPF and DTDR flood information went to available links. Consequently, with an increase in the number of ISLs composing the constellation, the communication overhead is two to three times higher. While the proposed scheme experiences a larger rate of increase in overhead as the number of satellites increases compared to the others, the absolute amount of communication overhead remains significantly lower. Compared to OPSPF, with the proposed scheme, the communication overhead is reduced by approximately 3.38%. This means that the proposed scheme becomes more effective in reducing communication overhead as the number of constellations increases.

### 5.3. End-to-End Delay in Routing Path Comparison

The end-to-end delay was compared for the three scenes in case of link failure. In this study, the ISL length was the only routing metric considered. Therefore, the propagation delay only includes end-to-end delay and is calculated as follows:

(8) $$d_{h}=\frac{e_{\mathrm{len}}}{c},$$

where $d_{h}$ is the propagation delay; $e_{\mathrm{len}}$ is the distance between the two satellite nodes; *c* is the speed of light in a vacuum. Then, the end-to-end delay $d_{p}$ in this simulation is calculated by

(9) $$d_{p}=\sum_{i=1}^{H_{c}}d_{hi},$$

where $H_{c}$ is the hop count of the path and $d_{hi}$ is the delay of the *i*th hop with a path.

**Figure 8:**
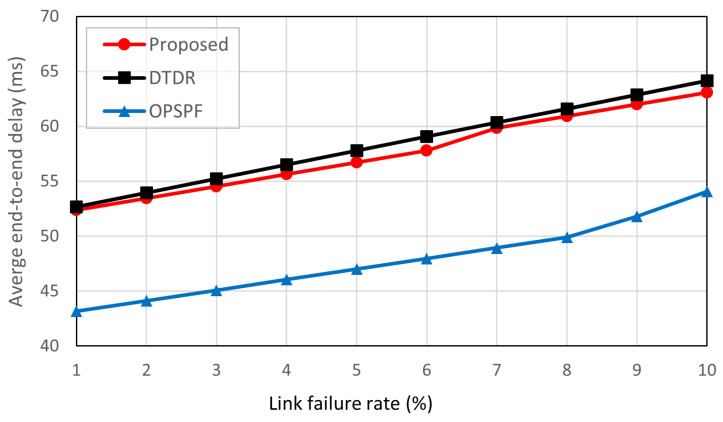
Average end-to-end delay according to link failure rate.

Figure 8 shows the average end-to-end delay based on the link failure rate. It is observed that OPSPF outperforms the proposed scheme and DTDR in end-to-end delay. In all link failure rates, OPSPF is 10 ms faster than the other algorithms. This is because the link-state routing scheme maintains the network in an up-to-date state through periodic information exchanges and flooding across the entire network. The flooded link information invokes the routing table to update the detour failure links.

**Figure 9:**
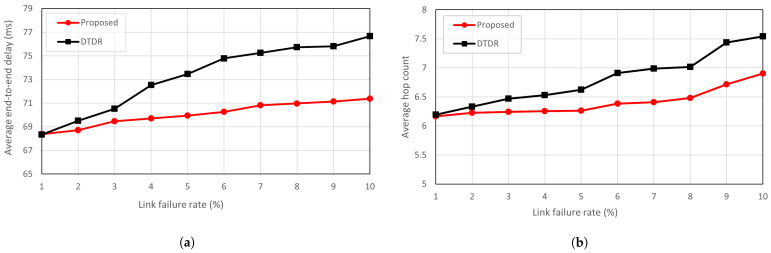
(**a**) Average end-to-end delay from detour path according to link failure rate; (**b**) average path hop count from detour path according to link failure rate.

The proposed scheme and DTDR use a hop-by-hop routing scheme, with only the direction of the next hop determined at any given moment. Consequently, link failures cannot be anticipated in advance, which leads to additional detour delays. However, as shown in Figure 8, the proposed scheme has a 1 ms lower end-to-end delay compared to the DTDR approach. Figure 9a further explains the results in Figure 8 showing that the increase in link failure rate results in the detour path of the proposed algorithm having a lower end-to-end delay. This is because the proposed scheme prioritizes using the shortest links in the MHR for detouring. As a result, the nodes within the MHR can detour the failed link without relying on it. OPSPF recalculates all routing tables whenever a link failure occurs to ensure an optimal detour path. Therefore, OPSPF is not included in the graph comparing the detour paths.

However, DTDR constructs a detour route by prioritizing the secondary direction as an alternative to link failure. The three-hop flooding approach does not know link failure information beyond three hops, and additional routing hops occur in paths exceeding three hops. As Figure 9b shows, there is a one-hop difference in the hop count between the proposed scheme and DTDR. Nevertheless, the proposed scheme selects the ISL with the optimal route within the MHR for detouring. Conversely, the DTDR approach cannot always detour to the optimal point because it uses the *n*-hop flooding technique, which introduces additional delays. This means that neither method shows a significant difference in the hop counts of the detour path. However, the proposed scheme generates a shorter detour route for a given hop count.

**Figure 10:**
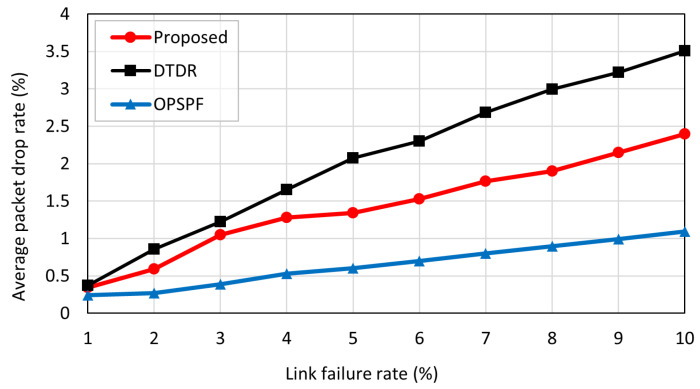
Average packet drop rate according to link failure rate.

The packet drop rate was further evaluated for the three schemes. Figure 10 shows the packet drop rate according to the link failure rate. The packet drop rate PDR was calculated as follows:

(10) PDR=(Number of packet dropped/Total number of packet sent)×100

OPSPF has the lowest packet drop rate. This is because OPSPF maintains up-to-date network information, ensuring that packets are routed without encountering link failure. However, packets that are transmitted during message flooding do not change their paths because of link-state changes. Consequently, the transmitted packets may encounter link failures and may be dropped. However, DTDR had the highest packet drop rate. This is because packets that encounter a link failure cannot take immediate action and are dropped from the network. The proposed scheme uses rapid detours to route packets that encounter link failure. As a result, it detours the link to reach its destination, which leads to a lower packet drop rate compared to DTDR. If the destination of a packet does not coincide with the intended destination, then the detour table cannot check for link failure information in the path. That means the packet drop rate of the proposed scheme is higher than that of OPSPF.

### 5.4. ISL Usage Rate for Flooding Comparison

**Figure 11:**
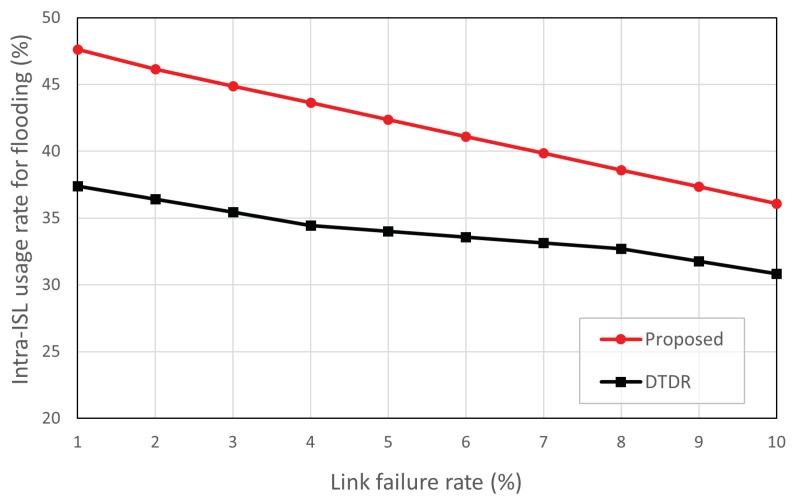
Average intra-ISL usage rate for flooding according to link failure rate.

The ISL laser module requires a PAT process for the link setup with LEO satellites, which results in a significant delay in ISL setup time. In particular, the performance in establishing a link can affect the end-to-end routing delay [27]. An intra-ISL connected within the same orbit can be maintained almost permanently. However, inter-ISL tends to be temporary because of the differences in distance and speed between satellites [25]. In this simulation, to handle unstable links in a more realistic environment, we evaluated the ratio of the ISL used in flooding. Figure 11 shows the ratio between intra-ISL for flooding. In Figure 11, the proposed scheme is only compared with DTDR. A comparison with OPSPF is meaningless because it uses all the network links for flooding.

Compared with DTDR, the proposed scheme consistently selects intra-ISL more frequently across all link failures. At a link failure rate of 1%, the proposed scheme uses approximately 10% more intra-ISL than DTDR. This is because the selective flooding path is determined based on the direction and geographical position of the failed link. In particular, link failures occurring in the Northern Hemisphere have selective flooding paths that primarily use intra-ISL. By contrast, DTDR floods all nodes within a predetermined *n* hop range, regardless of the position and direction of the failed links. The proposed scheme may vary depending on the geographical location of the satellite location. When considering situations with more traffic occurring in the Northern Hemisphere, the proposed scheme tends to utilize more intra-ISLs.

In an LEO satellite network routing design, relying on inter-ISL for flooding may not always ensure the transmission of link-state information because of link failures. This can result in routing loops or increased delays if the nodes fail to receive link-state information and are, thus, unable to choose the optimal detour path. However, the proposed scheme can enhance reliability during selective flooding for end-to-end transmission by increasing intra-ISL utilization compared with DTDR.

## 6. Conclusions

In this paper, we introduced a distributed detour routing scheme designed to minimize the communication overhead in LEO satellite networks. The proposed scheme detours failed links through rapid detours by leveraging a selective flooding algorithm to set up detour paths. After link recovery, the node checks the recovered link information in the detour table and reverts to the original path. We implemented the LEO constellation based on the Iridium constellation and compared it to two benchmark routing algorithms. The results show that the proposed scheme reduces the end-to-end delay compared with the *n*-hop flooding detour path. Selective flooding restricts the communication overhead to the MHR region. Finally, considering the realistic LEO satellite network, the proposed scheme uses more intra-ISL for information exchange.

The proposed distributed detour scheme was designed for polar constellations. The optimal detour point is determined in advance in polar constellations, but it is not determined in other constellations. Therefore, scalability will be considered to be further investigated in Walker-delta and mixed constellations. 

## Figures and Tables

**Table 1:** The parameters of the simulation.

Simulation Parameter	Value
The number of orbits	6
The number of satellites per orbit	11
Altitude	780 km
Inclination	$89^{\circ }$
ISL interface per satellites	4
Packet size	1 KB
Polar-boundary	$70^{\circ }$

**Table 2:** The amount of communication overhead for flooding by constellation.

Constellation [26]	OPSPF [7]	DTDR [11]	Proposed Scheme
Telesat (128)	1465	690	98
SpaceX (348)	12,525	9749	6139
OneWeb (588)	1,156,064	838,464	39,082

## Data Availability

The data generated during this current study are available from the authors upon reasonable request.

## Abbreviations

The following abbreviations are used in this manuscript:

LEO	Low Earth orbit
MEO	Medium Earth orbit
ISL	Inter-satellite link
RF	Radio frequency
PAT	Pointing, acquisition, and tracking
MHR	Minimum hop region
DTDR	Disruption tolerant distributed routing
OPSPF	Orbit prediction shortest path first
RT	Routing table
